# Exploratory Cohort Study of Associations between Serum C - Reactive Protein and Fatigue after Stroke

**DOI:** 10.1371/journal.pone.0143784

**Published:** 2015-11-24

**Authors:** Simiao Wu, Fiona Duncan, Niall H. Anderson, Annapoorna Kuppuswamy, Malcolm R. Macloed, Gillian E. Mead

**Affiliations:** 1 Centre for Clinical Brain Sciences, University of Edinburgh, Edinburgh, United Kingdom; 2 Department of Neurology, West China Hospital, Sichuan University, Chengdu, China; 3 School of Health and Life Sciences, Glasgow Caledonian University, Glasgow, United Kingdom; 4 Centre for Population Health Sciences, University of Edinburgh, Edinburgh, United Kingdom; 5 Sobell Department of Motor Neuroscience, University of College London, London, United Kingdom; Chiba University Center for Forensic Mental Health, JAPAN

## Abstract

**Background and Aim:**

Post-stroke fatigue is a common and distressing problem but little is known about its biological mechanisms. This cohort study was to investigate associations between C-reactive protein (CRP) and fatigue after stroke.

**Methods:**

Patients were assessed at one, six and 12 months after their stroke onset, with the Fatigue Assessment Scale, a case definition of post-stroke fatigue, Hospital Anxiety and Depression Scale, and daily step counts. Blood samples were collected at each assessment and the CRP level was determined by a standard CRP immunoassay. Cross-sectional associations between CRP and fatigue at each time point were determined by Pearson correlation coefficient and independent-samples t-test. Whether CRP levels at one month predict fatigue scores at six and 12 months was explored by multiple linear regression, with anxiety, depression, and daily step counts as covariates.

**Results:**

Sixty-five patients (mean age 67 years, 65% men) were included: 61 at one month, 49 at six months, and 41 at 12 months. CRP levels and fatigue scores were not associated at one month (*p* = 0.88) or 12 months (*p* = 0.56), but weakly associated at six months (r = 0.27, *p* = 0.04); however, this association was no longer significant (*p* = 0.14) after controlling for the effects of covariates. The CRP level was not associated with the fulfilment of case definition of post-stroke fatigue at any time points (all *p* > 0.05). The CRP level at one month was not a significant predictor for fatigue levels at either six months (*p* = 0.93) or 12 months (*p* = 0.78).

**Conclusions:**

There is insufficient evidence for the association between CRP and PSF in stroke patients. Future studies with larger sample sizes and controlling for potential confounders are needed to investigate whether this association exists.

## Introduction

Fatigue is a common and distressing problem for stroke patients, but there is no convincing evidence to support the use of any intervention for its treatment [1]. A better understanding of its mechanisms may help to inform the development of effective treatment strategies. Observational studies suggest that the presence of post-stroke fatigue (PSF) involves interactions of biological, psychological, behavioural and environmental factors [2]. Significant associations have been found between PSF and distressed mood [3] and reduced physical activity [4]. However, relatively little research has investigated biological mechanisms of PSF.

Fatigue in neurological disorders is often associated with both central nervous system and systemic inflammation [5]. Inflammation is an important pathophysiological response to stroke [6], which is characterised by the changed levels of peripheral inflammatory biomarkers, including C-reactive protein (CRP) [7]. A genetic study found that stroke patients with the single nucleotide polymorphism (SNP) rs4251961, an immune-regulating gene, were more likely to have higher fatigue scores [8]. This SNP is associated with a higher level of peripheral CRP in the general population [9]. However, findings on the direct association between CRP and chronic fatigue in patients with medical conditions were inconsistent across studies [10].

Two previous studies have explored the association between CRP and fatigue in stroke patients but reported no significant association [11, 12]. However, these two studies had small sample sizes of less than 50 patients and investigated only the association between acute phase CRP and PSF. Longitudinal studies in stroke indicated that CRP levels can be persistently elevated over several months after stroke [13]. This persistently elevated CRP might, in theory, contribute to the PSF in the longer term.

Therefore, this longitudinal study was to explore associations between serum CRP levels and fatigue during the first year follow-up after stroke, and to explore whether CRP levels at one month predict fatigue at a later stage after stroke.

## Methods

### Participants

The current study is part of a longitudinal cohort study to explore the nature history and clinical associations of fatigue in the first year after stroke [14]. In summary, from September 2009 to June 2011, we recruited 136 stroke patients within the first month after their stroke onset, who were admitted to hospital or seen in outpatient stroke clinic in Edinburgh, UK. Patients were assessed for clinical outcomes at one, six and 12 months after their stroke at the Clinical Research Facility, Royal Infirmary of Edinburgh. Due to practical and ethical issues, only 65 patients had blood samples collected, whom were included in the current study. The reasons for not collecting blood samples included that the patient was unable to attend the assessment at hospital, the patient refused to give blood samples, and the research nurses were unable to obtain blood samples after two attempts. Other exclusion criteria included subarachnoid haemorrhage, medically unstable due to another medical condition (including acute pneumonia, uncontrolled diabetes, and acute kidney injury), severe dysphasia, or severe cognitive impairment. Ethical approval was obtained from the Lothian Research Ethics Committee (09/S1103/1) and all patients gave written informed consent.

### Clinical assessment

Demographic and medical information was collected from medical records at recruitment. Baseline assessment included the Mini-Mental State Examination (MMSE), the National Institute of Health Stroke Scale (NIHSS), the Physical Activity Scale of Elderly (PASE) questionnaire for pre-stroke physical activity, and a question ‘Did you have a problem with fatigue before your stroke?’ The following clinical outcomes were assessed at one, six, and 12 months, respectively: a case definition of PSF [15]; the Fatigue Assessment Scale (FAS) [16]; the Hospital Anxiety and Depression Scale (HADS) [17]; and daily step counts (measured by an accelerometer, ActivPAL).

### Blood samples and CRP measurement

Blood samples were taken by research nurses at the Clinical Research Facility, during patients’ visit to Royal Infirmary of Edinburgh for clinical assessments at one, six, and 12 months. Samples were centrifuged immediately and stored in lithium heparin tubes at -80°C. The serum level of CRP was determined using a standard latex immunoassay (CRP Vario, Abbott Architect c16000). This standard protocol allowed a reportable range of CRP concentration from 0.2 to 320.0 mg/L, with a total coefficient of variation of 2.15% at a mean CRP concentration of 5.10 mg/L. We did not use the high sensitivity CRP assay because the local laboratory regulation (UK Accreditation Service) requires that the CRP concentration should be reported as whole numbers and the result of less than 1 mg/L be reported as ‘<1’ mg/L, where the sensitivity of the standard method that we used is comparable to that of the high sensitivity method.

### Statistical analysis

IBM SPSS 21 was used for statistical analyses. The CRP concentration less than 1 was replaced with 0.5 (i.e. half of the minimal identifiable values) for statistical analysis. Both the FAS scores and CRP levels were positively skewed thus were transformed using the 10-based logarithm, i.e. logFAS and logCRP. Little’s Missing Completely at Random test indicated that the data of clinical variables (including PSF case definition, logFAS, logCRP, HADS-anxiety scores, HADS-depression scores, and daily step counts) were missing completely at random (χ^2^ = 268.06, *p* = 0.44) and thus the missing data of these variables at one, six or 12 months were replaced using the Multiple Imputation (Automatic method, five imputations). The Multiple Imputation increased the sample size to 65 patients with valid data of clinical outcomes at all three assessments, which would give 80% power at a significance level of 0.05 (two-tailed) to detect a correlation coefficient of 0.34. This expected correlation coefficient is within the range of reported coefficients (r = 0.01 to 0.37) of the correlation between CRP levels and fatigue scores in previous stroke studies [11, 12].

Cross-sectional associations between logFAS and logCRP at each assessment time point were determined by the Pearson’s correlation coefficient (r). Independent-samples t-test was used to compare the logCRP levels between patients fulfilling and not fulfilling the PSF case definition. Cross-sectional correlations were further explored by multiple linear regression, with depression scores, anxiety scores, and daily step counts included as covariates. We included these covariates because they are significant correlates of fatigue in stroke patients [3, 4]. Because anxiety scores were shown to be a confounder for the cross-sectional association between CRP and fatigue levels, we performed post-hoc sensitivity analyses by excluding patients with a HADS-anxiety score of 11 or more as such score indicates the case of anxiety [17]. The sensitivity analyses were performed on the original data without imputation for missing data.

Furthermore, as the CRP level in the early stage after stroke is a useful predictor for stroke prognosis [7, 18], we used multiple linear regression to explore the predictive effect of CRP levels at one month on fatigue levels at six and 12 months, respectively. The above analyses were performed for each of the five imputations of the missing data and the pooled results of all five imputations were reported. For multiple linear regression, SPSS did not report the pooled results of adjusted coefficient of determination (adjusted R^2^) and F-ratio (F) for multiple imputations, thus we calculated these two pooled values using established methods [19].

## Results

### Descriptive data

A total of 65 patients who had at least one blood sample collected were included: 61 patients at one month, 49 patients at six months, and 41 patients at 12 months. Compared to the other 71 patients who were included in this longitudinal study but who did not provide any blood sample, these 65 patients were younger, physically more active before stroke, and with a lower proportion of diabetes, but no difference in other baseline characteristics (Table 1).

**Table 1:** Demographic and clinical characteristics of participants at recruitment.

Characteristics	Patients with blood samples collected (n = 65)	Patients without blood sample collected (n = 71)
Mean age (SD), years	67.3 (11.9)	72.2 (11.2)^a^
Male (%)	42 (64.6)	46 (64.5)
First-ever/Recurrent stroke	55/10	51/20
Ischaemic/Haemorrhagic stroke	62/3	65/6
Hypertension (%)	29 (44.6)	42 (59.1)
Diabetes (%)	5 (7.7)	18 (25.3)^a^
Pre-stroke fatigue (%)	24 (36.9)	33 (46.5)
Smoking (%)	14 (24.1), n = 58	14 (20.9), n = 67
Median NIHSS (IQR)	2 (1, 4), n = 63	2 (1, 4), n = 71
Median MMSE (IQR)	27 (26, 29), n = 52	27 (25, 28), n = 69
Median PASE (IQR)	120 (76, 173), n = 65	76 (44, 120)^a^, n = 70

SD: standard deviation; NIHSS: National Institute of Health Stroke Scale; IQR: interquartile range; MMSE: Mini-Mental State Examination; PASE: Physical Activity Scale of Elderly.

^a^ *p* < 0.05 (*p* values were calculated using Mann-Whitely U test for continuous variables and Chi-square test for dichotomous variables).

Table 2 presents the serum CRP levels and other clinical outcomes at each assessment of the 65 patients who provided blood samples.

**Table 2:** Clinical outcomes at one, six and 12 months after stroke.

	1 month (n = 61)	6 months (n = 49)	12 months (n = 41)
Median CRP (IQR) (mg/L)	2 (1, 4)	2 (0.5, 4)	2 (0.5, 4)
Median FAS (IQR)	23 (17, 29)	18 (16, 23)	20 (15, 24)
Fulfilling case definition of PSF (%)	17 (27.9)	7 (14.3)	4 (9.8)
Mean HADS-depression (SD)	6.5 (4.5)	4.9 (3.9)	4.5 (4.2)
Mean HADS-anxiety (SD)	5.2 (3.9)	3.7 (3.2)	3.4 (3.0)
Mean daily step count (SD) (×1,000)	5.788 (3.391), n = 40	5.618 (3.386), n = 39	6.354 (4.160), n = 32

CRP: C-reactive protein; IQR: interquartile range; FAS: Fatigue Assessment Scale; PSF: post-stroke fatigue; HADS: Hospital Anxiety and Depression Scale; SD: standard deviation.

### Cross-sectional associations between CRP levels and PSF

There was no significant correlation between CRP levels and FAS scores at either one month (r = 0.02, 95% confidence interval, 95% CI -0.23 to 0.26, *p* = 0.88) or 12 months (r = 0.09, 95% CI -0.16 to 0.33, *p* = 0.56). A weak (r = 0.27, 95% CI 0.03 to 0.48) but statistically significant (*p* = 0.04) correlation was found at six months. There were no significant differences in CRP levels between patients who fulfilled the PSF case definition and those who did not, at one month (t = 0.61, *p* = 0.54), six months (t = 1.84, *p* = 0.08), or 12 months (t = 0.31, *p* = 0.76).

A linear regression model was fitted to further explore cross-sectional associations between CRP levels and FAS scores, by including depression scores, anxiety scores, and daily step counts at each assessment as covariates. The CRP levels made no significant contribution to predicting FAS scores at any of these time points, whilst the anxiety score was the only significant predictor at all three time points (Table 3).

**Table 3:** Cross-sectional associations between C-reactive protein (CRP) levels and fatigue scores, controlling for anxiety, depression and daily step counts (n = 65).

	Model for logFAS (1 month)			Model for logFAS (6 months)			Model for logFAS (12 months)		
	B	SE of B	*p values*	B	SE of B	*p values*	B	SE of B	*p values*
(Constant)	1.220	0.043	<0.001	1.183	0.040	<0.001	1.259	0.044	<0.001
LogCRP	0.006	0.031	0.84	0.044	0.029	0.14	-0.038	0.036	0.31
Anxiety (HADS)	0.018	0.005	<0.001^a^	0.019	0.005	0.001^a^	0.013	0.006	0.03^a^
Depression (HADS)	0.010	0.006	0.09	0.004	0.006	0.53	0.021	0.009	0.03^a^
Daily step counts (×1000)	-0.007	0.005	0.18	-0.005	0.005	0.35	-0.014	0.004	0.007^a^
Adjusted R^2^	0.44			0.49			0.59		
F (*p* values)	13.62 (<0.001)^a^			16.39 (<0.001)^a^			24.03 (<0.001)^a^		

Blood samples were available from 61 patients at one month, 49 patients at six months, and 41 patients at 12 months. The regression analyses were performed for the whole cohort of 65 patients with Multiple Imputation for the missing data. LogCRP: logarithmic transformation of the serum concentration of C-reactive protein; HADS: Hospital Anxiety and Depression Scale; B: unstandardized regression coefficient; F: F ratio; R2: coefficient of determination; SE: standard error.

^a^ *p* < 0.05

Given the significant association between anxiety scores and FAS scores, sensitivity analyses were performed by excluding patients with the HADS-anxiety score of 11 or more at each assessment (Table 4). Significant associations were found between CRP levels and both the presence and severity of PSF at six months, but neither at one month nor 12 months (Table 4).

**Table 4:** Sensitivity analyses for the association between CRP and fatigue, by excluding patients with a Hospital Anxiety Depression Scale anxiety score of 11 or more.

Fatigue measures	1 month (n = 52)	6 months (n = 50)	12 months (n = 47)
FAS	r = -0.08, 95% CI -0.35 to 0.20 (*p* = 0.57)	r = 0.30, 95% CI 0.02 to 0.53 (*p* = 0.04)^a^	r = 0.07, 95% CI -0.22 to 0.35 (*p* = 0.68)
PSF case definition	t = 0.38 (*p* = 0.70)	t = 2.34 (*p* = 0.02)^a^	t = 0.63 (*p* = 0.53)

FAS: Fatigue Assessment Scale; PSF: post-stroke fatigue.

^a^ *p*<0.05.

### Relationships between CRP levels at one month and fatigue at follow up

The linear model regressed on the CRP levels, anxiety scores, depression scores, and daily step counts at one month after stroke explained 21% of the variance in FAS scores at six months and 24% variance at 12 months. However, CRP levels at one month made no significant contribution to predicting FAS scores at either six or 12 months (Table 5). The regression analyses were repeated by excluding the CRP from the model. Removing this variable had little effect on the model fit, as the adjusted R^2^ changed from 21% to 22% for FAS at six months (for the difference: F = 0.53, *p* = 0.47) and from 24% to 23% for FAS at 12 months (for the difference: F = 0.36, *p* = 0.25).

**Table 5:** Relationships between CRP levels at one month and fatigue scores at 6 and 12 months, controlling for anxiety, depression, and daily step counts (n = 65).

	Model for logFAS at 6 months			Model for logFAS at 12 months		
Predictors at 1 month	B	SE of B	*p values*	B	SE of B	*p values*
(Constant)	1.265	0.075	<0.001	1.278	0.074	<0.001
LogCRP	0.004	0.039	0.92	-0.016	0.056	0.78
Anxiety (HADS)	0.016	0.005	0.002^a^	0.017	0.006	0.007^a^
Depression (HADS)	-0.005	0.006	0.45	-0.005	0.008	0.99
Daily steps (×1000)	-0.008	0.008	0.38	-0.010	0.008	0.20
Adjusted R^2^	0.21			0.24		
F (*p* values)	5.34 (0.001)^a^			6.15 (0.0003)^a^		

Blood samples were available from 61 patients at one month, 49 patients at six months, and 41 patients at 12 months. The regression analyses were performed for the whole cohort of 65 patients with Multiple Imputation for the missing data. LogCRP: logarithmic transformation of the serum concentration of C-reactive protein; HADS: Hospital Anxiety and Depression Scale; B: unstandardized regression coefficient; F: F ratio; R2: coefficient of determination; SE: standard error.

^a^ *p*<0.05

## Discussion

This is the first study to investigate the relationship between CRP and fatigue over time after stroke. We found a weak but significant cross-sectional association between CRP levels and fatigue scores at six months after stroke, but this association was not significant at either one month or 12 months. CRP levels at one month made no significant contribution to predicting fatigue scores at either six or 12 months. Similar results were reported previously in smaller studies. A pilot study (n = 28) found no significant association between CRP levels and fatigue scores (r = 0.12, *p* = 0.55) in patients at a median time of 35 days (range 3 to 89 days) after stroke onset [11]. In a previous longitudinal study (n = 45), CRP levels during the acute phase of stroke were not significantly associated with fatigue scores at six, 12, or 18 months after stroke [12]. However, this longitudinal study found that another inflammatory biomarker IL-1β during the acute phase was significantly associated with fatigue levels at six months but not 12 or 18 months [12]. On the one hand, this is consistent with our findings that CRP was associated with fatigue levels at six months after stroke but not beyond this time point; on the other hand, this suggests that other biomarkers than CRP could also be indicators for the association between PSF and inflammation. The optimum biomarker for inflammation after stroke need to be identified in future studies.

Existing literature on PSF indicates that the development of ongoing fatigue after stroke is a temporal process and involves interactions of biological, psychological, behavioural and environmental factors [2]. Thus a possible explanation for the changed association between CRP and fatigue over time is that the development of fatigue at different stages after stroke is affected or even dominated by different factors. Qualitative studies suggest that during acute and subacute phases, PSF may be mainly attributed to stroke lesions and lifestyle changes following stroke [20]. However, the mechanisms how stroke triggers PSF are unknown and require future research. As stroke recovers over time, the effect of stroke itself on PSF may subsides and other factors, including but not limited to inflammation, take over the role. This may explain the lack of association between CRP and PSF at one month but a significant association at six months. We found a weak association between CRP levels and fatigue scores at six months, but this association disappeared after controlling the effects of anxiety, depression, and daily step counts, with anxiety as the only significant association. This suggests that the association between CRP and fatigue might have been confounded by anxiety. This hypothesis is supported by our findings of a higher correlation coefficient between CRP levels and fatigue scores after patients with high anxiety scores were excluded. Similar results were reported in a previous pilot study, where a significant association was found between CRP and PSF only after excluding patients with HADS scores of 28 or more [11].

Some researchers proposed the concept of cytokine-induced sickness behaviours [12], of which symptoms including depression, anxiety, fatigue, and social withdrawal could result from a same inflammatory origin [21]. In the current study, the effects of co-existing psychological factors on fatigue scores became more evident at 12 months than at one month and six months, where anxiety, depression, daily step counts but not CRP at 12 months were independent predictors for fatigue scores and the model explained 59% of the variance in fatigue scores. This may be explained by the findings from a longitudinal study that most observed inflammatory biomarkers (including CRP) decreased significantly by one year after stroke [22]. By this time, psychological factors such as depression and anxiety are still common in stroke patients. In the current cohort, fatigue scores were significantly associated with both depression and anxiety scores over time up to 12 months after stroke and higher anxiety scores at one month independently predicted higher fatigue scores at both six and 12 months [4]. However, relationships between inflammation and psychological factors in stroke patients are unclear, and whether they interact and how they affect PSF need to be investigated in future studies.

Although this is, to our knowledge, the largest study to explore the association between CRP and PSF, the statistical power of this study is limited. Although the correlation coefficient (r = 0.27) at six months was smaller than the pre-specified minimal significant correlation coefficient (r = 0.34), this correlation was statistically significant in the current study (which had a sample size n = 65 with a power of 60% to detect the significance of a correlation coefficient of 0.27). However, this needs to be confirmed in future studies with larger sample sizes. Another weakness of this study is that CRP data were not available for all patients in the cohort. Compared to patients who did not provide blood samples, patients who provided blood samples were younger, physically more active, and with less comorbidities, thus were more likely to attend hospital visits to provide blood samples. This might have led to some bias in the current study and the generalisability to the older and physically less active stroke patients would be limited.

## Conclusions

There is insufficient evidence for the association between CRP and PSF in stroke patients. Future studies with larger sample sizes and controlling for potential confounders are needed to investigate whether this association exists.
